# Body Image Disturbance and Body Dissatisfaction in Sisters of Adolescent Girls with Anorexia Nervosa: A Narrative Review and a Preliminary Study

**DOI:** 10.3390/healthcare14131987

**Published:** 2026-07-03

**Authors:** Elisabet Tasa-Vinyals, Queralt Tristany-Dalmau, Arturo Rodríguez-Rey, Albert Martínez-Pinteño, Mireia Mora-Porta, Maria Teresa Plana, Susana Andrés-Perpiñá, Elena Moreno, Esteban Martínez, Luisa Lázaro, Josefina Castro-Fornieles, Itziar Flamarique

**Affiliations:** 1 Department of Child and Adolescent Psychiatry and Psychology, Institut Clínic de Neurociències, Hospital Clinic of Barcelona, 08036 Barcelona, Spain; 2 Fundació de Recerca Clínic Barcelona-Institut d’Investigacions Biomèdiques August Pi i Sunyer (IDIBAPS), 08036 Barcelona, Spain; 3 Faculty of Medicine, University of Barcelona, 08036 Barcelona, Spain; 4 Department of Pediatrics and Neonatology, Manresa University Assistance Network, 08243 Manresa, Spain; 5 Department of Endocrinology and Nutrition, Institut Clínic de Malalties Digestives i Metabòliques, Hospital Clinic of Barcelona, 08036 Barcelona, Spain; 6 Faculty of Psychology, University of Barcelona, 08036 Barcelona, Spain; 7 Centro de Investigación Biomédica en Red de Salud Mental, CIBERSAM—ISCIII, 28029 Madrid, Spain

**Keywords:** body image, body disturbance, body dissatisfaction, siblings, anorexia nervosa

## Abstract

**Highlights:**

**What are the main findings?**

- Most sisters of adolescent girls with severe anorexia nervosa (AN) tend to considerably overestimate their body size, both generally and part-by-part, particularly calves, waist and chest, a pattern of disturbance. The observed pattern appears noteworthy when set against previously published normative and clinical findings, although direct comparison is limited by the absence of a matched control group. Though a minority of underestimators was detected, it was smaller than previously described in community samples of preadolescent and adolescent girls.
- Body image disturbance and body dissatisfaction did not correlate in our sample, though the former correlated directly with physical activity and the latter correlated directly and considerably with depressive symptoms and self-oriented perfectionism.
- Neither body image disturbance nor body dissatisfaction seemed to significantly relate to actual corporeality, at least as measured using standard anthropometric methods such as body mass index.

**What are the implications of the main findings?**

- Several risk factors converge in young sisters of girls with severe AN, including shared genetics, shared environment, female sex/gender, exposure to a potentially traumatic event such as serious disease of a sister, and ongoing neurodevelopment. Findings raise the possibility that sisters of adolescents with severe AN may represent a group in whom body-image-related vulnerability warrants closer longitudinal study, particularly girls experiencing their sister’s disease at younger ages, as they show greater trait anxiety and might tend to show greater body image disturbance.
- While further research including larger and more diverse samples (particularly including brothers) is needed to confirm and contextualize these results, this preliminary study offers findings that warrant further investigation. Sisters of patients with AN may represent an important group for future research on body-image vulnerability and broader mental health risk.

**Abstract:**

**Background:** Body image disturbance (BID) involves a distorted perception of one’s body, whereas body dissatisfaction (BDS) reflects negative affective evaluation of body appearance. Both are central features of eating disorders such as anorexia nervosa (AN), but their expression in siblings of affected individuals remains underexplored, particularly in younger populations. **Methods:** This cross-sectional preliminary study included 53 full sisters of adolescent patients with severe AN treated in a tertiary hospital. Participants underwent anthropometric assessment and completed standardized measures of BID, BDS, physical activity, eating-related attitudes, perfectionism, anxiety, and depressive symptoms. **Results:** Participants exhibited marked body size overestimation (mean relative BID = +45.2%), with 64.2% classified as high overestimators and 3.8% as underestimators. Overestimation was most pronounced in the waist, chest, and calves. BID was not significantly associated with BDS or with anthropometric measures, including body mass index. BDS showed significant positive correlations with depressive symptoms and self-oriented perfectionism, whereas BID was positively associated with physical activity. No significant associations were found between BID or BDS and age, socioeconomic status, or birth order. **Conclusions:** Sisters of adolescents with severe AN show substantial perceptual distortion of body size without corresponding levels of body dissatisfaction, suggesting partial independence between perceptual and affective components of body image. These findings identify a potentially vulnerable group and highlight the need for longitudinal studies to clarify mechanisms and inform preventive strategies.

## 1. Introduction

Body image problems in siblings of patients with anorexia nervosa (AN) represent an important yet underexplored area, as these individuals share familial and sociocultural environments that may contribute to body image in several complex ways. Emerging evidence based on a systematic review and a literature review on sibling experiences with eating disorders [1,2], and a systematic review on psychopathology and other mental health challenges experienced by siblings themselves [3], suggests that siblings might constitute a vulnerable group, with possible sex/gender differences indicating that sisters may be more likely to internalize body image dissatisfaction, while brothers may present more subtle or distinct patterns of concern. However, though all of them mention the issue, none of the mentioned reviews focus on body image specifically. Therefore, prior to introducing the design and results of a preliminary study on body image problems in a sample of sisters of underage patients suffering from severe AN, in this section we first present a narrative review on the topic. Unlike other types of review (scoping or systematic reviews), a narrative review offers a flexible, qualitative method for synthesizing and critically interpreting the existing literature, typically relying on a broad, non-systematic search and an integrative analysis of diverse sources to identify key themes and research gaps [4,5]. This approach is particularly useful for complex or under-researched topics such as sibling experiences in particular areas related to eating disorders such as body image concerns, although the interpretive nature and lack of standardized methodology characterizing narrative reviews may introduce subjectivity and limit reproducibility. Accordingly, this section aims to introduce basic body image terminology and briefly map current knowledge on body image problems in siblings of individuals with severe eating disorders like AN, focusing on their characteristics and underlying mechanisms while integrating them in the broader context.

### 1.1. Body Image, Body Image Disturbance and Body Dissatisfaction

Body image is part of an individual’s self-concept and can be defined as a complex construct comprising a person’s perceptions, thoughts, feelings, and behaviors towards or related to their own body. Body image discordance is the discrepancy between self-estimated and objectively measured body size. Body image disturbance (BID), sometimes referred to simply as body disturbance, occurs when a significant body image discordance exists, usually in a context characterized by a persistent and impairing negative affect focused on one’s own body and/or physical appearance that has behavioral and bodily consequences [6,7,8,9,10]. Some authors refer to body image discordance as the perceptual component of BID (perceived-actual discrepancy), in contrast with a second, affective component (perceived-ideal discrepancy), which is also known as body dissatisfaction (BDS) [11].

That being said, two important remarks shall be made: (a) everyone has a body image, definable as a perceptive-affective phenomenon that will naturally develop over time (mostly during neurodevelopmental stages, e.g., childhood and adolescence) against the backdrop of cultural and societal norms, and (b) body image problems, including body shape concerns, BDS and eventually BID, are not exclusive of pathological conditions such as eating disorders or body dysmorphic disorder but rather a set of universal highly gendered phenomena socially rooted in aesthetic violence, which is a form of gender-based violence [9,12,13,14,15,16,17,18,19,20]. However, as is discussed later, the presence of severe and functionally impairing forms of body image problems, usually including severe BID, is a core feature of many eating and body image disorders and required for diagnosis of AN, for instance.

Importantly, given its psychological burden and its association with severe pathological conditions such as eating disorders, BID can be modified by specific preventive and therapeutic strategies, e.g., dissonance-based, theatre-based, media literacy-based, and even videogame-based interventions [21,22,23,24,25,26]. Preventively speaking, such interventions can reduce the risk of developing eating, feeding and body image disorders through several pathways, including decreasing drive for thinness and disordered eating, and increasing media literacy and critical thinking, besides reducing the economic and social costs of BDS and appearance-based discrimination [21,22,24,25,27,28,29,30,31]. On a therapeutic level, once a disorder exists, addressing BID with similar dissonance-based interventions can also potentially improve this core symptom and therefore contribute to recovery in the context of a wider therapeutic intervention, such as family cognitive behavioral therapy (F-CBT) supported by other specific interventions when needed, e.g., trauma-informed, bodily centered practices or desensitization and reprocessing therapies [27,32,33].

Research on BID and other body image problems in community samples leads to heterogeneous results and sometimes complex conclusions. A systematic review of BDS and sociocultural messages related to the body among preschool children including 16 studies found that 20–70 percent of the children (depending on the method of assessment) showed BDS with no significant gender differences and in both directions (children wishing for a thinner or a larger body), and that parents’ comments and behaviors were the main influence on body image development in children at this stage, aged 0 to 6 [34]. Another review focused on children aged 6 to 11 found results similar to those reported in adolescents and adults, with desire for a thin body already established as the normative experience, and with a clear influence of gender, age, body mass index (BMI), race, sociocultural pressures, and self-concept on children’s body image concerns and even early eating disturbances [35]. A study on Korean high school students found that approximately 40 percent of them had BID, both boys and girls; however, the distortion pattern was reversed, with most boys underestimating their actual body size (26.5 percent of the total sample) and most girls overestimating it (25.3 percent of the total sample) [17]. Another study including girls aged 10 to 15 found that postmenarcheal girls were more prone to BDS and distorted eating compared to girls who had not yet experienced their first period, and that greater levels of BID after menarche correlated strongly with other psychologic variables such as BDS and overall negative affect [19].

### 1.2. Factors Associated with BID and Other Body Image Problems in Healthy and At-Risk Populations

Several factors have been associated with a poor body image or BID. Family influences on BID have often been examined, generally pointing to mothers and sisters as important pressure sources potentially leading to body image and eating concerns, with transcultural differences that, when observed, tend to end up affecting women and girls in Western families more negatively, at least as long as migration processes are kept out of the equation [12,36]. Regarding peer and school environment influences, academic achievement and weight control have been identified as significant factors associated with BID in adolescents of both genders, along with physical exercise in the case of boys and socioeconomic status in the case of girls [17]. A systematic review and meta-analysis on social comparison in social media, body image concerns and eating disorder symptoms found a significant, moderate and direct correlation between engagement in social comparison practices in social media and altered body image and eating patterns, significantly moderated by gender [37]. The centrality of gender and other cultural contextual factors in the complex relationship between social media exposure and physical measurements, media internalization and body image problems has been emphasized by other studies, also in males [14,38,39,40,41]. Interestingly, a study reports that individuals struggling with BID and other related body image concerns prefer same-sex clinicians and researchers for purposes related to body assessment and discussion, while healthy individuals generally do not show a preference [13].

The relationship between BID and physical activity has been repeatedly explored. A systematic review on physical activity and body image in adolescents (aged 10–18) based on 28 studies concluded that a negative association exists between these variables in healthy community subjects, with more physically active individuals being less likely to develop BID. However, the authors highlight the existence of potential bias in the examined research, particularly regarding the use of suboptimal BID and physical activity assessment methods in study designs that might not capture potentially relevant mediation effects of other variables [42]. In the same line, a study of high school students found that individuals showing BID were significantly more likely to be sedentary [18]. Conversely, another study assessing a group of preadolescents and adolescents found weak associations between physical activity and body image that tended to disappear when adjusting for BMI [43].

Studies based on the specific population of elite athletes complement the previous ones. A systematic review focused on athletes integrating results of 31 studies concludes that the evidence available is complex and inconclusive, though it seems clear that a subgroup of athletes (female, young, and whose sports are associated with leanness) seem more likely to develop body image problems and also eating disorders [44]. An umbrella review on the same topic, based on 24 systematic reviews of which 10 included meta-analysis, concluded that elite athletes had an increased risk of disordered eating and eating disorders but at the same time were at lower risk for body image concerns, when compared to non-athlete controls [45]. This study basically identified the same risk factors as the previously mentioned one: female gender, leanness-associated sports, and added experiencing career changes as an extra factor, but in this case for disordered eating, since according to this review physical activity—also on an elite sports level—protects from body image concerns.

### 1.3. BID in Eating, Feeding and Body Image Disorders: The Case of AN

AN is a severe psychiatric–metabolic disorder classically characterized by the following: (a) severe dietary restriction and other compulsive behaviors aimed at keeping body weight below age- and sex-appropriate levels compatible with bodily functions and health; (b) overwhelming fear of weight gain; and (c) cognitive impairments such as poor insight and distorted body image [46]. AN is the deadliest of eating, feeding and body image-related disorders and typically starts during late childhood or adolescence, with girls being at higher risk (female-to-male ratio is estimated to be at least 10:1) [47]. Despite long-term, ongoing controversies in diagnostic criteria of AN [48], body image disturbance (or simply body disturbance, BID) remains a core part of current diagnostic criterion C in the DSM-5, as was in previous editions, e.g., DSM-IV-TR [46,49].

It is currently controversial among experts whether BID in the context of AN is a result of a primary perceptive impairment or rather a cognitive-affective phenomenon related to negative self-esteem and a self-evaluation unduly influenced by body weight and shape, as mentioned in DSM [46]. BID tends to improve with re-nutrition during the first phases of AN recovery, suggesting a pure perceptual basis. However, we know that patients with AN at a particular point of their recovery can accurately assess bodies of other individuals despite showing great BID when assessing their own [32]. Similarly, a study examining brain activity linked to self-image assessment in AN patients and controls revealed that the latter showed activation in the middle frontal gyri, insula, precuneus and occipital regions when assessing their own image, whereas the former showed heightened amygdala activity, an area linked to emotional information processing. However, when AN patients viewed images of others, their brain activity patterns resembled those of the control group. This seems to support the idea that, although body image is created through a basis of perceptual (mostly visuo-spatial) mechanisms, its affective and cognitive dimensions are products of the intercession of emotional and cognitive (beliefs) information coming from other neuroanatomic regions [8,50,51].

A significant and well-known familial predisposition exists in the development of eating and body image disorders, which are characterized by abnormal eating attitudes and behaviors aimed at changing body weight/shape even in ways that threaten health or even life, serious body image concerns or distortion, an overrepresentation of body weight and/or shape in self-concept, and often an impaired insight. Over the years, several studies have shown heritability estimations among the highest for mental disorders, as well as a remarkable familial aggregation between cases and their first-degree relatives, particularly mothers; with fewer studies involving sisters, and even less including brothers [3,26,52,53,54]. Besides shared genetics, and unlike the case of mother-daughter dyads, sisters are usually girls of similar age who share familial, school, and social environments at a given historical and cultural moment. In other words, their shared environment is allegedly larger and potentially more impactful. While many studies have focused on siblings’ experiences and emotional management challenges throughout the illness of their affected sisters, research on specific mechanisms and pathways underlying sisters’ vulnerability to eating and body image disorders, including BID, is scarce [3]. To the best of our knowledge, only one study systematically engages in BID assessment of siblings of patients with AN from a mostly perceptual standpoint, concluding that they seem to be relatively preserved in terms of BID despite some interesting findings in brothers of patients compared to male controls [55]. Another study psychometrically explores body shape concerns, more linked to the affective component of BID, in siblings of patients with AN, failing to find significant differences between siblings and controls [56]. These results are not fully consistent with other evidence underlining higher prevalences of several disorders and/or syndromes linked to BID in siblings of patients with AN, including also concepts related to BID such as interoceptive awareness [52,57,58,59].

On a risk factor level, certain personality traits are known to increase risk of AN, typically including perfectionism, obsessivity and neuroticism or trait anxiety [51,60,61]. When examining AN patients and their healthy sisters regarding these traits, the existing literature suggests that significant differences could be premorbid, with sisters tending to be more cognitively flexible, less perfectionist and less self-concerned (trait anxiety), which arguably makes them more independent and mirrors their family to a lesser extent than patients [56].

### 1.4. BID and Other Problems Related to Body Image in the Specific High-Risk Population of Sisters of Individuals with AN

Despite robust evidence of siblings being at a higher risk of AN and other related disorders and symptoms, the studies regarding pathways and susceptibility to disease in this population describe complex and sometimes unexpected results. As far as we are aware, two reviews exist on the experience of having a sibling with an eating disorder and the potential psychosocial impact on the sibling family subsystem and on the siblings as individuals [1,2]. One of these reviews is a systematic review [2]. In addition, a single systematic review exists focused on the translation of these potential psychosocial challenges into clinical psychopathology measurable by quantitative means, including categorical (diagnoses, syndromes) and dimensional (symptom) assessments in siblings of patients with AN [3]. As previously mentioned, this recent systematic review identifies only one study specifically examining BID in siblings of patients with AN, while a few more include assessments of phenomena somehow related, such as BDS in the context of distorted eating and/or eating attitudes assessment, usually pointing to negative effects on siblings, particularly in sisters [55].

In the two more phenomenological reviews, sibling participants coincide in underlining the relevance of themes such as premorbid familial attitudes towards food and body image (e.g., body shaming, diet culture), and whether such attitudes change upon diagnosis of AN in a family member [1,2]. In general, siblings tend to experience AN as a challenge for their mental well-being and the family system’s well-functioning, including the sibling subsystem, as defined in Bowen’s Family Systems Theory [62]. However, some siblings report a positive or protective effect of exposure to symptoms of AN, such as emaciation and BID, since witnessing their consequences might trigger rejection towards diet culture and body image concerns. However, such protective, transformative and/or resilient responses seem limited to cases where exceptionally good practices regarding siblings’ role in therapeutic and familiar settings have taken place, paired with certain education and family communication styles that might boost resilience in siblings [1,2,3,55,56]. Understanding the specific yet diverse experiences and challenges faced by siblings in the context of familial AN is crucial for designing tailored interventions and support mechanisms aimed at mitigating psychological distress and promoting resilience among siblings coping with the systemic sequelae of this pervasive disorder, also in terms of body image problems [1,2,3,55].

### 1.5. Objectives, Hypotheses and Potential Applications

Objectives:

This preliminary study aims to contribute to current knowledge by systematic, objective, standardized, and clinician-made assessment of BID in a group of sisters of patients with child- and/or adolescent-onset severe AN admitted to a specific hospital unit for moderate to severe eating disorders in underage patients.

The sisters’ assessments also include sociodemographic, basic anthropometric, and psychometric evaluation of variables strongly related to BID, such as eating and body image-related attitudes and behaviors, pattern and level of physical activity, affective and anxiety symptoms, perfectionism, and trait anxiety, to explore their relationship to BID.

Hypotheses:

i. Main

(a) Relatively high prevalences of BID and/or BDS will be generally found in sisters overall.

(b) BDS will be positively related to BID and, since it is supposed to reflect the affective component of body image, BDS might mediate BID and affective phenomena (trait anxiety, depressive symptoms).

ii. Exploratory

(c) A subgroup of sisters might show an atypical BID pattern potentially protective from eating and body image disorders, e.g., lower levels of BID (accurate estimation) or even inverse pattern BID (underestimation).

(d) Certain sociodemographic (age), anthropometric (BMI), lifestyle (physical activity) and/or psychological (perfectionism, trait anxiety, depressive symptoms) variables will be associated with BID and/or BDS in this population.

In this study, BMI is defined as weight in kilograms divided by height in meters; physical activity is measured by a self-administered validated questionnaire commonly used to assess frequency and intensity of physical activity in children and adolescents; and perfectionism, trait anxiety and depressive symptoms are defined as the scores obtained in self-administered, specific, validated psychometric tools measuring these variables (see further detail in the next section).

Translationally contextualized aim of this research:

Remarkably, the results obtained in this preliminary study may guide future research and analysis of larger and/or complementary samples (e.g., samples including both sisters and brothers) and eventually inform and encourage the design and implementation of specific strategies to prevent BID in sisters/siblings of individuals already affected by AN, understood as a critically important high-risk population.

## 2. Materials and Methods

### 2.1. Participants and Procedures

This cross-sectional preliminary study analyzed data obtained from 53 full sisters of patients suffering from severe AN treated in the Eating Disorders Unit of the Department of Child and Adolescent Psychiatry and Psychology of Hospital Clinic in Barcelona, Spain. This unit treats underage patients diagnosed with severe eating disorders from the area of Barcelona and is part of the Spanish public health system. This means that all underage patients in clinical need are eligible for treatment in the unit, for as long as clinically appropriate and at no direct cost for the families. In Spain, access to healthcare is universal, regardless of citizenship status or past/current employment situation or tax-paying status. Patients/families always have the option to receive treatment within the public health system, and they have the choice to receive it within the private health system as well. Public healthcare centers are sectorized and receive patients from the nearby area, though specific units for severe cases such as ours have bigger influence areas: for instance, our unit receives eligible patients from almost every administrative sector in the city of Barcelona and also parts of neighboring cities such as Badalona or Sant Adrià del Besòs, areas that differ substantially in their socioeconomic characteristics.

We followed a two-step sampling procedure. The first one was incidental: the study was designed and took place in our Eating Disorders Unit, since it was designed by our team and a multicenter approach did not seem optimal for a preliminary study. The second sampling step is described in the following paragraph.

Upon approval by the hospital’s ethics committee, clinical records of all patients with a diagnosis of AN who had received treatment in the unit during the past 2 years, regardless of their current clinical status and/or previous referrals, were reviewed in order to identify those who had full sisters meeting eligibility criteria. All potentially eligible sisters and their families were informed about the study and offered voluntary, anonymous participation. Brothers were excluded due to the preliminary nature of the study, in an attempt to maximize statistical power as sample size was relatively small. We set a limit of the past 2 years for logistic reasons, since patients having received treatment prior to that could arguably be more difficult to contact and/or more reluctant to return to the hospital facility they had probably already been discharged from. Since ours is a unit for underage severe patients with eating disorders, in case a patient is not discharged before they turn eighteen, they are referred to the adult mental health network to continue treatment and therefore stop being treated in our unit.

Once a participant was recruited, written informed consent was obtained by the sister and, if underage, from a parent or legal guardian. Sociodemographic and basic clinical records were obtained upon recruitment. Families were sent a REDCap link by email and asked to complete psychometric assessments online [63]. A single face-to-face assessment was scheduled for each sister during which a nurse obtained anthropometric data (height, weight, waist and hip width) and a trained psychiatrist or psychologist tested BID.

Inclusion criteria for sisters were as follows:

I1: Full sisters aged 8 to 25 of patients diagnosed with AN (probands) treated in our unit, regardless of the type, clinical features or outcomes, with no limitation as to the number of sisters recruitable per proband.

I2: Willingness to participate and provide oral and written informed consent.

Exclusion criteria for sisters were as follows:

E1: Severe disability preventing completion of the evaluation protocol.

E2: Inability or unwillingness to provide written and oral informed consent.

Importantly, a past or current diagnosis of an eating disorder or other psychiatric comorbidity was not considered an exclusion criterion.

This preliminary paper presents the results of the first 53 sisters recruited, between 2023 and 2024.

### 2.2. Variables and Measurements

Sociodemographic data: age, socioeconomic status according to the Hollingshead index [64], sister order (older or younger than the AN proband), and mental health records were obtained via interview.

Anthropometric data: height, weight, waist width, and hip width were measured by a trained nurse and not shared with participants. BMI was calculated as weight in kilograms divided by height in square meters (kg/m^2^).

**Figure 1:**
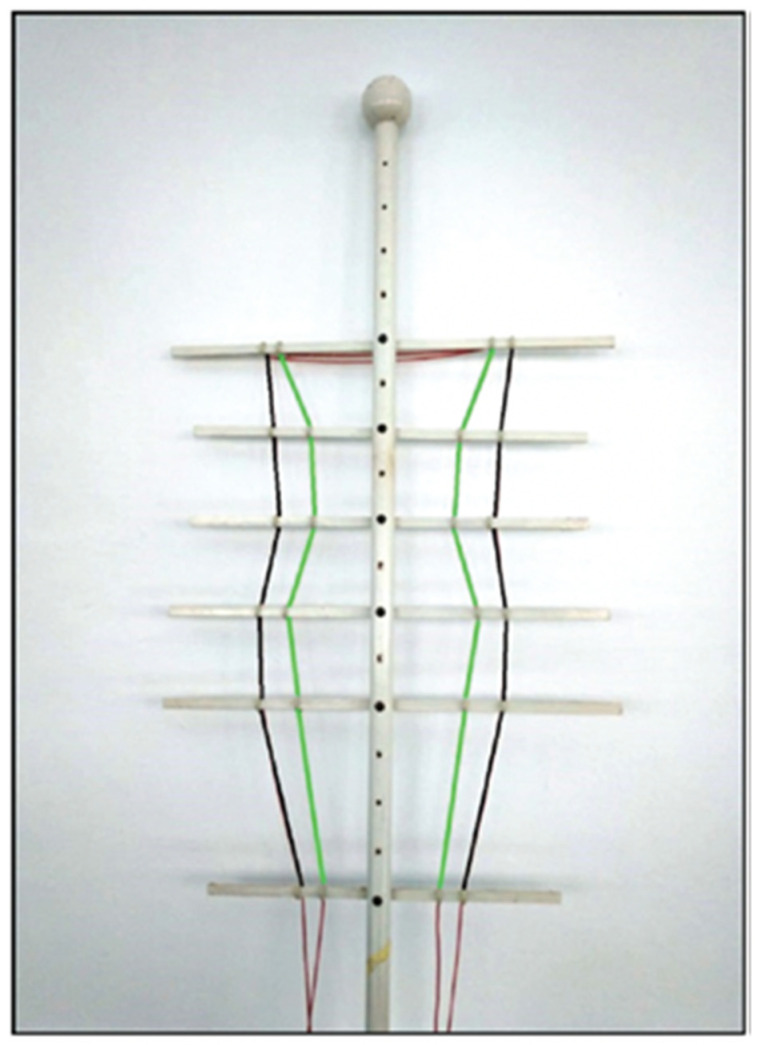
Self-estimation of the own body size of an adolescent girl obtained with the SBDA. The girl’s actual body silhouette (based on objective measurements) is shown in green, whilst her subjective perception is represented in black. The difference between the two represents BID and can be expressed absolutely (in cm) and relatively (in percent). Source: Plana et al. (2024), reproduced with permission [32].

Subjective Body Dimensions Apparatus (SBDA) [7]: Several methods exist to assess BID and BD [65]. In this study design, we chose to combine an objective, perceptual measurement of BID with psychometric approaches to BD. The SBDA is a tool for objective assessment of the perceptual component of BID originally developed in Spain in 1998. Similar to the silhouette test, the SBDA remains used in both clinical and research settings to the present time [32]. It consists of a 190 cm high cylindrical bar vertically mounted on a base, which can be adjusted to the participant’s height (Figure 1). Along the bar, 6 sticks are positioned perpendicularly, representing body parts: shoulders, chest, waist, hips, thighs, and calves. Each of the bars is provided with 2 sets of adjustable rings and strings that can be moved along the bar to reflect estimated and real body measurements. The sticks are each equipped with a measuring tape on the back, not visible to the participant. For this task, participants are requested to stand still facing the SBDA, at approximately 2 m distance, and asked to think of the apparatus as a sort of mirror reflecting their full body. The evaluator stands behind the apparatus and slowly moves the first set of rings (2 rings per bar) symmetrically along each of the bars representing the different body parts, starting with shoulders and ending with calves. The participant is instructed to verbally sign the point at which they think the rings appropriately capture their body size for each body part. After repeating the process in each of the 6 bars, a human silhouette is formed representing the participant’s perceived body shape. The second set of rings can be used later to represent real measurements obtained from the participant for each body part. Both silhouettes can thus be simultaneously represented, visually compared and/or tape-measured for arithmetic comparison, obtaining the rough difference in centimeters and the relative difference in percentual points between real and perceived body part sizes. In both cases, positive results (higher than zero) indicate that the subject overestimates their body size, while negative results (lower than zero) indicate underestimation. Results close to zero indicate that the subject represents their actual body size accurately. In our study protocol, participants were introduced to the task in a gamified way, and during the session with the evaluator, only the first set of rings (perceived body dimensions) was used. BID was calculated later by researchers by comparing these dimensions with real body dimensions obtained for each participant with a tape measure at a different point of the session. The SBDA was initially validated in 85 patients with AN and 472 controls aged 12–18, with satisfactory results [7]. Normative profile charts for the general population have also been obtained with a sample of 802 females aged 11–24 [66].

Eating Disorder Inventory-3 (EDI-3) [67,68] in Spanish [69], the latest version of Garner’s inventories for the screening and assessment of eating disorders among youth [70]. This 91-item self-report questionnaire consists of the following subscales:

- Eating disorder-specific scales, interpretable as risk of eating disorders: drive for thinness (DT), bulimia (B), and body dissatisfaction (BDS).
- Psychological trait scales, interpretable as risk of general psychopathology and/or mental distress: low self-esteem (LSE), interpersonal alienation (PA), interpersonal insecurity (II), interoceptive deficits (ID), emotional dysregulation (ED), perfectionism (P), asceticism (A), and maturity fears (MF).

The psychological trait scales can be grouped into the following composite scores or indexes: Ineffectiveness Composite (IC), Interpersonal Problems Composite (IPC), Affective Problems Composite (APC), Overcontrol Composite (OC), and General Psychological Maladjustment Composite (GPMC).

Physical Activity Questionnaire for Children or Adolescents (PAQ-C/PAQ-A) [71] in Spanish [72,73]. The PAQ is a self-report instrument designed to measure the frequency and intensity of physical activity in children aged 8 to 14 (PAQ-C) or adolescents aged over 14 (PAQ-A). Its 9 items assess different dimensions of physical activity, such as participation in sports, active playing, and sedentary behaviors, during weekdays and weekends. The total score is calculated as the mean score of all items and can range from 1 to 5 points. Partial scores based on particular items can also be used to reflect specific physical activity patterns (e.g., frequency and intensity of physical activity of any kind during the week). Reviews and meta-analyses comparing subjective versus objective measurements of physical activity and updated psychometric properties of physical activity questionnaires for children and adolescents guided our choice of this instrument [73,74,75]. The Spanish version of the PAQ has shown satisfactory reliability and validity results [72]. Psychometric studies show that the PAQ, particularly the PAQ-A, can be used to classify adolescents as active or inactive following international recommendations, with a cut-off point of 2.75 above which a subject can be considered physically active [76].

Child-Adolescent Perfectionism Scale (CAPS) [77] in Spanish [78,79]. The CAPS is a 22-item self-administered questionnaire based on a bidimensional conceptualization of perfectionism. It consists of 2 subscales: self-oriented perfectionism (SOP) with 12 items and scores ranging from 1 to 60, and socially prescribed perfectionism (SPP) with 10 items and scores ranging from 1 to 50. Higher scores reflect a higher presence of the trait. In Spain, a 2004 study administered the CAPS to a group of 113 adolescents from the general population and 71 adolescents with AN, demonstrating good internal consistency (Cronbach’s alpha for the general population = 0.85) and one-week test-retest reliability (r = 0.80). In 2019, another study with a community sample of 1809 primary school children obtained similarly satisfactory levels of reliability and stability of 0.91 and 0.73, respectively.

State-Trait Anxiety Inventory (STAI) for sisters aged 16 and over, or an adapted version for those 15 or younger (STAIC) [80,81,82,83]. This classical self-report psychometric tool, developed by Spielberger and colleagues, is commonly used to measure both state (momentary) and trait (long-standing) anxiety levels in individuals. It originally consisted of 2 separate inventories designed to evaluate temporary anxiety experienced in specific situations (state anxiety) as well as the general tendency to perceive situations as threatening (trait anxiety). Trait anxiety is conceptually similar to personality traits such as neuroticism or a tendency for obsessive thinking. For this study, only the trait anxiety inventory was used. A total score is obtained by combining the scores associated with each multiple-choice answer given by the subject in each item. Scores are inverted if necessary (inverse items). Total scores can range from 0 to 60 points, and highest scores imply higher levels of trait anxiety. The Spanish versions of both instruments obtained satisfactory psychometric results in various validation studies [84,85,86].

Beck Depression Inventory (BDI) for sisters aged 17 or over, or the adapted version Child Depression Inventory (CDI) for those 16 or younger [87,88]. These self-report instruments, created by Beck and Kovacs respectively, assess the existence and severity of depressive symptoms, e.g., sad or irritable mood, anhedonia, trouble sleeping, eating or concentrating, negative cognitions, thoughts or intention of self-harm. Scoring works similarly to that with STAI/STAIC, and total scores can range from 0 to 63 points (from 0 to 53 points in the case of the CDI), with higher scores implying more and/or more severe depressive symptoms. Scores above the cut-off point of 20 are usually considered deserving further assessment by clinical interviewing. The Spanish versions of both inventories obtained satisfactory psychometric results in several validation studies [89,90,91].

### 2.3. Ethical Considerations

Given that some of the sisters/families could feel pressure to participate due to their AN-affected daughter being currently treated in the unit, the right to withdraw from the study whenever they wished without any consequences for them or for the patient was stressed. Participants were also offered the possibility to refuse to take any psychometric tests that they did not feel comfortable with, as a minority of families expressed concerns about sisters being exposed to questionnaires explicitly referring to disturbed eating and/or body image concerns. In such cases, participants/families were asked about the exact reasons for their refusal, including whether they had experienced/observed any specific difficulty regarding eating behavior, body image, or emotional well-being. Distress was not monitored during SBDA in a standardized manner, though participants were fully aware that they could decide to withdraw from the study at any time, including during testing. Additionally, the clinician-researchers involved were trained to detect any type of distress noticeable during testing and to intervene accordingly.

Regardless of family concerns, sisters were not shown their own anthropometric measurements, since there is currently no clear evidence excluding the possibility that exposure to such data in vulnerable individuals might increase the risk of BDS, disordered eating, or other potentially harmful outcomes. All cases were included in statistical analysis, regardless of whether they had refused to take any of the tests or not.

The ethical issue of balance between data completeness and participant safety was carefully considered in the study design, and researchers’ choice was to prioritize participant protection whenever any concern was raised.

Arguably because of these ethical considerations, along with the strong bond between patients/families and professionals developed during lengthy, intensive treatments such as the ones required by severe AN, the refusal rate was low (<10 percent) and mostly due to logistic difficulties as reported by the families.

### 2.4. Statistical Analysis

Data were described and analyzed according to the nature of each variable (quantitative or categorical).

Primary descriptive analysis:

Descriptive values for quantitative (mean, standard deviation, and range) and categorical variables (frequencies) were obtained. To analyze BID values, we used descriptive parameters applied to absolute disturbance (difference between estimated and actual body size, in centimeters) and relative disturbance (difference expressed as a percentage), for overall body size (mean of all body parts considered) and by body parts.

Exploratory, correlational and subgroup analyses:

Correlation coefficients were used to assess relationships between quantitative variables (e.g., BID and perfectionism), while Student’s T or ANOVA analyses were applied to compare means between groups defined by categorical variables (e.g., BID and physical activity expressed as an ordinal variable). Some analyses were complemented by recoding quantitative variables into categorical ones based on reasonable subject grouping, e.g., BID categories (negative disturbance, slight disturbance, considerable disturbance) and age categories (preadolescents/early adolescents, late adolescents/young adults).

Subgroup analyses were also performed with the main outcome (BID) between sisters with and without a diagnosis of eating disorder and between those who had taken the full evaluation set versus those who had refused to undergo certain measurements, to approach the issue of missingness being eventually related to the very constructs being measured.

For each statistically significant inter-group comparison, effect size measures η^2^, ε^2^, ω^2^ fixed, and ω^2^ random effect were calculated (point estimates and statistical significance via 95 percent confidence intervals).

Due to the exploratory nature of the study and the sample size, non-parametric test alternatives, multiple comparisons, and multilevel modelling were not considered.

The level of statistical significance was set at *p* < 0.05.

All statistical analyses were performed using SPSS 30 for MacOS.

## 3. Results

### 3.1. Sample Description

The sample included 53 sisters with a mean age of 16.13 years (standard deviation, SD = 2.89) and an age range between 10 and 24. The 53 sisters belonged to 46 sets of sisters, of which seven included the proband plus two sisters, and the rest included the proband and a sister. Thirteen sisters (24.5 percent of the sample) were twins with the proband, and there was an additional set of two twin sisters not involving the proband. Overall, 28.3 percent of the sample were products of multiple births. Twins excluded, 80 percent of the sisters were younger than the proband, and 20 percent were older. The socioeconomic status of the sample, according to the Hollingshead index, was mid–high (mean score 58.38, SD 11.27). A total of 11.3 percent of the sisters had a past or present formal eating disorder diagnosis, as reported by themselves and/or their families (that is, a diagnosis made by a licensed clinician). Reported diagnoses were AN (9.4 percent) and BN (1.9 percent). All of them were in total or partial recovery, though most were still receiving some sort of outpatient care at the time of the assessment, including psychotherapy sessions for issues not directly related to the eating disorder. The sample obtained high scores in perfectionism (particularly self-oriented), trait anxiety, and depressive symptoms, with 22.6 percent of the sample scoring above the BDI/CDI cut-off of ≥20 points. Table 1 presents a full sample description, including the number of participants who consented to provide data for each measure. Nine participants/families (16.9 percent of the sample) refused to answer the PAQ and the EDI-3 due to expressed concerns regarding eventual triggering of eating, body image, or traumatic difficulties. However, none of them reported having noticed any actual difficulties in their particular cases. No cases of distress during the administration of any of the tasks, including the SBDA, were detected.

### 3.2. Body Image Disturbance

We found a mean global (mean of six body parts considered) absolute BID of +11.44 cm (SD 8.78) and a mean global relative BID of +45.24 percent (SD 33.38). When excluding sisters with a reported history of an eating disorder, mean BID absolute and relative values did not significantly change and were actually marginally higher: +11.58 cm (SD 9.02) and +46.2 percent (SD 34.55), though the difference was not statistically significant. Absolute and relative BID values for each body part and the mean of all body parts were also very similar when comparing sisters with and without a history of an eating disorder, and no statistically significant differences were observed (all *p* values > 0.05, overall and by body parts). Similarly, BID values of sisters who had skipped any of the measures (particularly EDI-3 and PAQ) due to fear of triggering or revictimization were also statistically compared with those who had taken full part in the study, with analogous results: no statistically significant differences were observed (all *p* values > 0.05). Table 2 shows absolute and relative BID data for each body part, including minimum and maximum values observed and range.

The sample was divided into three groups based on BID: those who underestimated their body size (negative BID score) or underestimators, those who slightly overestimated it similarly to expected in the general population (relative BID of 0–30 percent) [66,92] or neutral/slight overestimators, and those who overestimated it to a greater extent (relative BID > 30 percent) or great overestimators. Results are displayed in Table 3.

Globally, only 3.8 percent of the sample were underestimators, while the vast majority of the sisters overestimated their body size, and 64.2 percent overestimated it to a greater extent than usually observed in the general female population. Shoulders were the most accurately estimated body part, while in the cases of chest, waist and calves, great overestimation occurred in over 70 percent of cases. In each body part except for shoulders, the largest group was the one of great overestimators, that is, the group who overestimated by far (>30 percent) the actual dimensions of their body. The minority of underestimators obtained negative BID mean scores of between −10 and −20 percentage points in each body part (that means that they estimated their body parts to be between 10 and 20 percent smaller than they actually were). In contrast, the majority of great overestimators believed their different body parts to be considerably bigger than they actually were, particularly waist and calves (more than 70 percent bigger).

### 3.3. Body Dissatisfaction

BDS descriptives are shown in Table 1, as measured by the homonymous scale of the EDI-3 (mean score 12.11, SD 8.88), which corresponds to a T score of 45 (non-clinical range) for female adolescents. Remarkably, nine (16.9 percent) participants/families declined to answer the EDI-3, mostly out of fear that exposure to questions about eating and body image disturbances might trigger either such problems or potentially traumatic memories of the sick sister’s disease. When excluding sisters with a reported history of an eating disorder, mean BDS scores did only slightly change (mean score 11.51, SD 8.10), and, similarly to the case of BID, were actually mildly lower. When comparing sisters with and without a history of an eating disorder, no statistically significant differences were observed in BDS (F = 1.59, *p* = 0.214), nor in any other scale of the EDI-3 except for drive for thinness (F = 4.22, *p* = 0.046), though effect size was marginal and only statistically significant when calculated by simplest effect size measure η^2^ (point estimate = 0.091, 95% CI [<0.001, 0.272], remaining non-significant with other more complex measures arguably more appropriate for this particular intergroup comparison (ε^2^, ω^2^ fixed and ω^2^ random effect). None of the other variables included in this study showed any statistically significant difference between sisters with and without a history of eating disorder.

### 3.4. Body Image Disturbance and Body Dissatisfaction

BDS did not significantly correlate with global BID (mean of all body parts), neither when BID was absolutely (r = 0.164, *p* = 0.287) nor relatively expressed (r = 0.157, *p* = 0.308). Similar results were obtained when assessing correlations between BDS and part-by-part absolute or relative BID, and no statistically significant correlation was observed. When dividing the sample by BID underestimators, slight overestimators, and great overestimators) did not statistically differ on BDS (mean = 8, SD 1.41; mean = 10.64, SD 8.7; and mean = 13.14, SD 9.24, respectively; *p* = 0.562).

### 3.5. Psychological Correlates

However, in the exploratory analyses, we observed differences between BID-based categories in emotional dysregulation (ED, EDI-3) (F = 3.412, *p* = 0.043, η^2^ size effect, point estimate = 0.143, 95% CI [<0.001, 0.315], rest of size effect point estimates below significance level). Mean (SD) emotional dysregulation values were 13.5 (6.36) for underestimators, 3.79 (SD 3.1) for slight overestimators, and 5.88 (5.46) for great overestimators. Given the small sample size, particularly of the group of underestimators, and the size effect point estimate results, this result should be interpreted cautiously even on a purely statistical level. None of the other variables considered in this study differed significantly between sample subgroups based on BID. BID as a quantitative variable did not show any significant correlation with perfectionism, trait anxiety, or depressive symptoms.

Conversely, BDS did show a significant positive moderate–strong correlation with depressive symptoms as measured by the BDI/CDI (r = 0.604, *p* < 0.001) and also a significant positive moderate correlation with self-oriented perfectionism (r = 0.427, *p* = 0.004), while no significant relation was observed between BDS and socially prescribed perfectionism nor trait anxiety.

### 3.6. Physical Activity, Sociodemographic Characteristics and Anthropometry

BID significantly correlated with physical activity as measured by total PAQ score in a positive and moderate way (r = 0.425, *p* = 0.004). The correlation between BID and physical activity expressed as the number of active days in a week did not achieve statistical significance (F = 2.39, *p* = 0.07).

No significant correlation was observed between BID and age in years, socioeconomic status, sister order, waist width, hip width, or BMI. Similarly, we did not find any significant relationship between BDS and physical activity, age in years, socioeconomic status, sister order, waist width, hip width, or BMI. Categorical analyses of BMI were not performed given the sample distribution, with the large majority of the participants falling within the category of normal weight. Given the underage condition of the sample, analyzing weight and height (components of BMI) as independent variables was pointless.

Given the developmental heterogeneity of a sample of youngsters undergoing neurodevelopment, we split the sisters into two groups: those aged below 16 (n = 26, 49.1 percent) and those aged 16 or older (n = 27, 50.9 percent). Mean BID was +48.09 percent (SD 36.94) in the younger subsample and 42.49 percent (SD 30) in the older subsample, though the difference was not statistically significant. Regarding BDS, the younger group obtained a mean score of 11.18 (SD 7.88) versus a mean score of 13.05 (SD 9.87) for the older group, a difference that did not reach statistical significance either.

None of the variables considered in this study showed significant differences by age group except for trait anxiety, as measured by STAI/STAIC, which was significantly lower in sisters aged above 16 (mean = 28.4, SD 8.62) than in sisters of younger age (mean = 40.39, SD 9.5) (F = 20.12, *p* < 0.001, ε^2^ size effect, point estimate = 0.298, 95% CI [0.082, 0.478].

Table 4 presents an hypotheses-guided summary of results.

## 4. Discussion

In this preliminary study, we cross-sectionally studied a sample of 53 biological sisters of underage patients with severe AN recruited from 46 families in order to assess if they presented BID, BDS, to what extent, if and how BID and BDS were related in this sample, and whether these body image problems connected to certain psychological variables linked to eating- and body image-related disorders’ etiopathogenesis according to the literature, as well as to physical activity and sociodemographic and anthropometric variables.

Our sample was mainly composed of sisters younger than the proband with a high proportion of twins (28.5 percent of the whole sample), remarkably higher than the one found in the general population, which is estimated to be 2–2.5 percent. The age range was 8 to 25, though the mean sample profile was adolescent, with a normative weight, not particularly physically active, and composed mostly of psychologically healthy girls on the screening level, despite a remarkable percentage of 11.3 percent having a past or present history of eating disorder (mostly AN) formally diagnosed by a licensed clinician. This prevalence, several times higher than the one observed in the general population, is consistent with the literature pointing to an approximately ten-fold increase in risk of AN for first-degree relatives and the high heritability of the disease [53,54,93].

Our main objectives were to assess BID in sisters of underage patients suffering from severe AN in a clinician-made, systematic, objective and standardized way and to examine whether and how BID and BDS were linked in this population. Exploratory objectives included testing the relationship between BID/BDS and distorted eating and body image-related attitudes and behaviors, pattern and level of physical activity, depressive symptoms, trait anxiety, perfectionism, and certain sociodemographic variables (e.g., age, sister order).

Regarding our hypotheses, formulated against the backdrop of the current literature broadly and extensively reviewed and narrated in the introduction section in the form of a narrative review [4,5], we expected to find the following results: (a) relatively high prevalences of BID and/or BDS will be generally found in sisters overall; (b) BDS will be positively related to BID and, since according to the most robust current literature BDS seems to correspond to the affective component of body image while BID is thought to correspond to the perceptive component, BDS could plausibly mediate BID and affective phenomena (anxiety, depressive symptoms); (c) based on two high-quality reviews recently published [1,2], a subgroup of sisters showing an atypical BID pattern potentially protective from eating and body image disorders, e.g., lower levels of BID (accurate estimation) or even inverse pattern BID (underestimation) might be identifiable in the sample; (d) certain sociodemographic (age), anthropometric (i.e., BMI), lifestyle (physical activity) and/or psychological (perfectionism, trait anxiety, depressive symptoms) variables will be associated with BID and/or BDS in this sample.

### 4.1. Body Image Disturbance: Sisters Showed Substantial Body-Size Overestimation

Our results are consistent with previous studies in terms of variability in sibling response/reaction to severe diagnosis of AN in another sibling [1,2]. However, though we did find sisters who did not distort body image or who did so only slightly or in a similar way than girls and women from general population, and also a minority of sisters (<10 percent) who actually underestimated the real dimensions of their own body, in our study the majority of sisters greatly overestimated their body size globally and by body parts, particularly waist and—in contrast with some previous studies [7,66,94]—also calves, a phenomenon that is discussed later in this section. We reported a remarkable degree of BID in the sisters, both in absolute and in relative terms, which persisted after removing those with a past or present history of eating disorder even though they were all in full or partial recovery at the time of the assessment. Globally speaking, the girls in our sample overestimated their own body size by more than 11 cm and estimated their bodies to be over 45 percent larger than they actually were, a figure that went up to 62 percent larger than actual body size in the case of great overestimators, who were the majority of the sample. Looking at ranges, we can see that certain subjects in this at-risk sample overestimated their body parts by more than double their size (BID of >+100 percent), which are levels of disturbance usually observed in patients with severe AN.

In contrast, a small yet considerable number of sisters underestimated the size of some part of their body. Though this finding has been reported previously in a minority of healthy girls and women, in our sample it was far less common than in other studies based on community samples (approximately 4 percent versus 14 percent) [17]. In any case, body size underestimation is relatively rare in girls and, particularly, in female adolescents and women [7,66,94,95,96]. Despite the fact that underestimation of mean body size was only observed in a minority of one in twenty sisters, one third of our sample underestimated the size of at least a part of their body at some point during the test. A possible explanation for this might point to the existence of a certain protective component to the experience of a severe eating disorder in a sister, by which some sisters might change the way they look at and think of their bodies to differ from the previous and/or usual, consistent with the previous literature [1,2,55]. We observed this phenomenon of underestimation more frequently at the shoulder level, whereas it remained rare in the waist and hips. From our point of view, these differences observed between body parts could reflect qualitative differences ascribed to body parts in the cultural aesthetic model for female bodies [9,97]. Thus, with a greater aesthetic pressure put on the central areas of the female body, it might be easier to tend to overestimate these parts even in subjects whose personal history with a sister with a severe eating disorder led to a counterstrategy of underestimation, let alone the majority of sisters who engaged in other coping strategies involving validating the discourse of the sister with AN at least partially or even trying to follow her steps out of admiration or for instrumental reasons (i.e., benefits of the AN symptoms such as parental and/or social attention and validation).

If we examine our data in the context of previous studies on BID carried out with adolescents with AN and controls, we find that mean BID in our sample of sisters of patients with AN is, as most of the previous literature concludes, closer to values obtained by controls than those obtained by their sick relatives [7,32,55,94,95]. Importantly, this does not mean that sisters are any less at risk of mental health challenges and/or disorders, since a recent systematic review has shown that psychometric, dimensional and/or symptomatic assessments might not appropriately detect psychopathology in siblings of patients with AN if not completed by clinical interviews [3]. In this sense, our preliminary study design presents the advantage of a clinician-made, live, dynamic, and systematic evaluation of BID, unlike some other studies that work with self-assessed BID questionnaires or tasks. Notably, though, BID (including underestimators and overestimators by more than 30 percent of real body size) was twice as commonly observed in our sample of sisters than in adolescent samples in the general population (roughly 80 percent versus 40 percent). Similarly, the great majority of our sample were overestimators compared to approximately 25 percent of adolescent girls in a community-based study [17].

### 4.2. Body Image Disturbance: Overestimation Appeared Across Several Body Parts

Despite sisters’ results resembling more those of controls and/or girls from the general population than those of girls affected by serious eating disorders, our study detects differences regarding the body parts participants tend to overestimate the most. If we look at previous studies carried out in our center, the parts that patients with AN overestimated the most were chest, waist and hips, qualitatively similar to controls but to a significantly greater extent [7,32,41,66]. In contrast, in our sample, although the greatest values of BID were observed in the waist, chest and hips fell to the third and fifth position respectively regarding degree of distortion, while calves (only after waist) tended to be remarkably overestimated. In fact, in our sample, a lower degree of BID was observed in shoulders compared to the previously mentioned studies, while data for hips were very similar to what was observed among both patients with AN and controls. In contrast, BID observed in chest, waist, thighs and calves in our sample is even greater than BID previously reported for these body parts in the group of patients with AN. One possible explanation for these results might be that sisters are already familiar with the disease and consciously avoid focusing their attention on the body parts traditionally more problematic for girls and women (avoidance). In contrast, however, our data suggest that sisters’ overall body perception might remain distorted, and we observe BID to be even more pronounced in parts of the body other than the ones that patients with AN typically distort. This could reflect qualitative differences in body image problems experienced by, on the one hand, healthy young women and girls from the community, and, on the other hand, women and girls at high risk or with direct experience of a severe eating disorder such as AN during neurodevelopment, which is associated with an increased vulnerability. Notably, taking into account the sociohistorical context, some of the previous studies referenced in this paper were published decades apart from ours, and aesthetic ideals for girls and women might have changed in the current context of light, non-radical feminism considerably influenced by constructionism and other intellectual standpoints that disregard bodily materialism.

### 4.3. Body Image Disturbance and Body Dissatisfaction Were Not Significantly Associated in This Sample

Regarding the connections between BID and other conceptually related variables, the failure to find significant associations between BID and key variables assessed by the EDI-3—including BDS—in this study is perhaps one of its most remarkable findings. The fact that, in this preliminary sample, perceptual and affective body-image dimensions appear to show different patterns of association might suggest that BDS could be not merely the affective component of body image, but a variable related to BID in more complex ways, since it seems that at least certain subjects can have remarkable degrees of BID and not be particularly or consistently dissatisfied with their bodies on an affective level. Variables such as general psychological adjustment could explain this fact and have actually been mentioned in the previous literature on siblings of patients with eating disorders specifically [1,2,55,98]. Our results suggest that BID might present independently from BDS, by alternative mechanisms including (but not limited to) BID showing previously and/or independently of abnormal eating behaviors and symptoms, which would have to be predominantly perceptive pathways (possibly neurocognitive phenotypes, for instance). Besides an eventual distinctive perceptual profile among sisters, other possible explanations for these findings could include mere reflection of developmental heterogeneity across the age range as a sample characteristic (since this is one of the few published studies including a young sample in their neurodevelopmental period, aged 8 to 25), or the effect of limited statistical power associated with a small preliminary cross-sectional study. However, if confirmed in future studies including larger samples and longitudinal designs, this finding could support a need for BID and BDS to be assessed and/or addressed separately in certain populations, i.e., sibling-focused screening and research, besides adding valuable knowledge that helps understand the complex mechanisms and functions of body image in general or other specific populations [9]. These ideas need to be interpreted cautiously due to the small sample size and the characteristics of the sample (high-risk first-degree relatives of patients with severe AN, all undergoing neurodevelopment during the experience with the sister’s disease and the assessment).

Interestingly, however, BID categorically expressed showed a weak statistically significant link to another affect-related scale of the EDI-3, ED measuring emotional dysregulation, in an unexpected sense: individuals more accurately estimating their body size seemed to have the lowest emotional dysregulation values, while individuals underestimating their body size scored more than three times higher than the former in emotional dysregulation, whereas greater overestimators of body size showed intermediate mean emotional dysregulation values.

### 4.4. Body Dissatisfaction Related More Clearly to Depressive Symptoms and Self-Oriented Perfectionism

Importantly, in our sample, BDS significantly correlated in a direct way and with moderate-strong intensity with depressive symptoms and moderate intensity with self-oriented perfectionism. These correlations support the idea of BDS being a key affective cornerstone of body image problems and, perhaps, the connection between perfectionism and depression, which might occur without the perceptual component (BID) in some cases. Interestingly, BDS was not significantly related to socially prescribed perfectionism nor even *actual* corporeality, supporting the idea of an affective pathway to distress/disease relatively independent of perception, either perception of reality or perceptual disturbance. The non-significance of BMI regarding BID and BDS in this sample conceptually could arguably support post-body-centered and fatphobic approaches to eating- and body image-related problems. In any case, these complex issues definitely require further research endeavors beyond the scope of this preliminary study.

In this paper, we use specific and precise terminology taken from disciplines such as discursive analysis, critical studies or intersectional sex/gender theory. In this context, we refer to corporeality (or corpomateriality) or bodily reality in the sense of measured body dimensions, i.e., BMI, understood as apparatuses of bodily production. Interested readers who are not familiar with these academic fields might want to explore basic works by authors such as Donna Haraway [99] and Nina Lykke, for instance [100].

### 4.5. An Exploratory Association with Physical Activity

Regarding the relationship between physical activity and BID, which was mentioned in the narrative review and included in our hypotheses, we shall start off by saying that our sample was not particularly physically active, making comparisons with specific high-risk populations such as elite athletes challenging. However, in this particular sample and using a specific methodology (PAQ), we observed a significant, direct and moderate correlation between the level of physical activity and BID, opposite to findings pointing to physical activity being negatively associated with BID in adolescents despite the well-known association of certain sports with an increased risk of eating disorders [18,42]. Since in this population, the psychological meaning of physical activity may be as important as its frequency or intensity, we remain cautious about findings about body image problems and PAQ scores. As previously mentioned, PAQ captures activity level, but it does not necessarily capture motivation or emotional function and has not been specifically designed to capture and/or distinguish health-oriented activity, appearance-related exercise, anxiety-linked movement, compensatory behavior, or body monitoring, aspects that are crucial in people diagnosed with or at risk for eating disorders. Thus, this finding needs to be interpreted as exploratory and tributary of further research for full understanding.

### 4.6. Anthropometric Measures Did Not Appear to Explain the Body-Image Findings

The findings do not seem to support the idea of anthropometry or bodily reality being a possible mediator or confounding factor between BID and other variables such as physical activity either, as suggested by certain previous studies carried out in preadolescents and adolescents [43]. This is consistent with the idea that all bodies can develop an eating disorder, including a severe case of AN, which is a key idea in the current literature on eating disorders that led to the specific mention of atypical AN in the DSM-5, a first step toward a possible paradigm change in the future of categorical psychopathology classifications [101,102,103,104].

### 4.7. Strengths and Limitations

This paper has several strengths and limitations that shall be acknowledged. To the best of our knowledge, this is the first study involving children, adolescents, and young adults to analyze how a sister’s diagnosis of AN at such ages might impact body image from a quantitative point of view and, specifically, from the complementary point of view of BID and BDS. We used an objective, comprehensive, specific, and robust measurement tool for BID in children, adolescents and youngsters, thus facilitating study replication and comparison with other samples.

The inclusion of sisters with current or past diagnoses of eating disorders is, in our view, another strength of the study design, since it increases external validity. Similarly, unlike many sibling studies previously published, the present study did not limit participation to a single sister per proband. A special emphasis was placed on ethical issues given the particular vulnerability of the sample (underage sisters of children and adolescents with severe AN), likely leading to an increased prevalence of missing data. However, despite inter-group comparisons showing no statistical differences, these methodological decisions call for a cautious interpretation of results as they might be linked to limitations such as possible within-family clustering and missing-data bias. Readers should bear in mind that sisters from the same family arguably support a significantly greater weight of shared environment, e.g., socioeconomic context, exposure to diet culture within the household, and exposure to the same proband’s illness. Future research might overcome this potential limitation with multilevel modeling. Notably, the proportion of sisters diagnosed with an eating disorder in our sample is fully concordant with the previous literature, which arguably increases the credibility of this study.

In our view, the main limitation of this preliminary study is the lack of a control group, which precludes optimal comparison between sisters and equivalent subjects. It should be underlined that all comparisons included in this paper are indirect comparisons with community and clinical populations. Besides the relatively small sample size limiting generalizability, another major limitation is the non-inclusion of brothers. Moreover, the cross-sectional design does not allow for causal inferences, and the study design did not include potentially relevant data such as actual and desired body composition and/or psychometric measurements of specific body image preoccupations. In this regard, it should be stressed that physical activity was measured with the PAQ, a general questionnaire intended for healthy children and adolescents, leading to a limited interpretation potential in terms of motivation and image-related characteristics of physical activity patterns. This limitation is partially overcome by the evaluation of such aspects in other questionnaires, particularly EDI-3. However, it shall be noted that 16.9 percent of the sample refused to complete these two particular questionnaires (PAQ and EDI-3) due to expressed concerns regarding risk of triggering or revictimization. This might be interpreted as proof that sisters/families identified physical activity as a potentially triggering area, despite the general nature of the PAQ. Other potential limitations include possible selection bias, limited generalizability beyond a tertiary hospital context, unavailability of sample-specific reliability coefficients, and shared-method variance.

### 4.8. Directions for Future Research

Future research should aim to address these limitations by working with controlled, larger and more diverse samples (particularly including male siblings), employing longitudinal designs, and exploring sex/gender differences in the impact of eating disorders on siblings of patients with AN, as well as potential sex/gender pathways to body image distress/disease. The current findings support replication, longitudinal follow-up, and better characterization of risk markers. Future research may perhaps, in the mid- and long-term, help inform the development of targeted, appropriate and timely prevention and intervention strategies for at-risk individuals if such interventions prove useful and necessary.

## 5. Conclusions

Sisters of patients with AN tended to overestimate their body size. The majority (64.2 percent) of the sample overestimated their mean body size by more than 30 percent, while a minority of 3.8 percent underestimated it. Globally speaking, sisters overestimated their own body silhouette by more than 11 cm and estimated their bodies to be over 45 percent larger than they actually were. The majority of greater overestimators perceived their body size to be 62 percent larger than it actually was. Some sisters overestimated their body size by more than 100 percent, to an extent that is usually observed in clinical settings among patients with eating disorders. Relatively greater overestimations were observed in the areas of calves, waist and chest, a pattern that appears noteworthy when set against previously published normative and clinical observations in both healthy and eating disorder-affected girls and women, although direct comparison is precluded by the absence of a matched control group. The proportion of underestimators observed among sisters was considerably inferior to those described in the general population and did not exceed 10 percent in any body part.

When indirectly compared to previously published data, sisters in this sample did not score significantly higher than the norm in BDS. Interestingly, no statistically significant correlation was observed between BID and BDS, suggesting the hypothesis that, at least in certain circumstances, one might occur without the other rather than reflecting different components of the construct body image (perceptual and affective, respectively). Moreover, both parameters seemed to be independent from actual body size, as reflected by the lack of correlation with anthropometric variables such as BMI.

In contrast, regarding exploratory analyses involving psychological correlates, BDS significantly correlated with body depressive symptoms (direct correlation, moderate-strong) and self-oriented perfectionism (direct correlation, moderate). No significant correlation was observed between BID/BDS and socially prescribed perfectionism, trait anxiety, drive for thinness, age, socioeconomic status, or sister order. BID/BDS results did not significantly change after removing sisters who had also developed an eating disorder from the sample, which represented over one in ten, a figure in accordance with the previous literature. Despite no significant correlations with age, sisters aged under 16 showed significantly higher trait anxiety scores in this sample, an exploratory finding that allows generating the hypotheses that this feature might decrease over time and place girls who witness their sister’s experience with AN at a younger age at a higher risk of psychopathology. Despite not being significant, younger sisters presented greater BID but lower BDS. All of these exploratory findings warrant further research.

In this sample, physical activity measured with a questionnaire that does not include motivation showed a direct correlation with BID in sisters, a result not supported by the previous literature on the relationship between physical activity and body image, though challenging to interpret outside the context of elite athletes and, particularly, without the motivation component in the context of at-risk subjects for eating disorders.

Limitations of this preliminary study shall be overcome by designs including control groups and larger and more diverse samples, particularly including brothers of patients with AN. Other particularly interesting future research designs could include further structured clinician-made psychopathology assessments, longitudinal designs that allow follow-up, and mixed-methods approaches that allow inclusion of qualitative and/or phenomenological information in combination with rigorous clinical and diagnostic assessments by licensed clinicians.

## Figures and Tables

**Table 1:** Sample description.

	N	Mean	Standard Deviation	Minimum–Maximum (Range)
BMI (kg/m^2^)	48	20.2	2.88	14–27 (13)
Height (cm)	53	160.1	6.18	150–175 (25)
Weight (kg)	48	52.1	9.3	34–76 (42)
Waist width (cm)	47	68.2	7.81	35–86 (51)
Hip width (cm)	47	77.82	9.17	41–97 (56)
Physical activity level, total score (PAQ)	44	1.58	0.37	
Physical activity pattern, last week (PAQ)	44		%	Valid %
		All or most of free time sedentary	22.6	27.3
		1–2 times/week	28.3	34.1
		3–4 times/week	24.5	29.5
		5–6 times/week	5.7	6.8
		7 or more times/week	1.9	2.3
Perfectionism, self-oriented (CAPS)	45	40.07	10.33	21–58 (37)
Perfectionism, socially prescribed (CAPS)	45	25.62	9.09	10–45 (35)
Trait anxiety (STAI/STAIC)	46	34.4	10.83	12–56 (44)
Depressive symptoms (BDI/CDI)	45	14.78	12.3	0–39 (39)
Drive for thinness, DT (EDI-3)	44	6.32	8.1	0–28 (28)
Bulimia, B (EDI-3)	44	4.84	6.29	0–24 (24)
Body dissatisfaction, BDS (EDI-3)	44	12.11	8.88	0–35 (35)
Low self-esteem, LSE (EDI-3)	44	7.91	5.93	0–20 (20)
Interpersonal insecurity, II (EDI-3)	44	10.75	5.65	0–22 (22)
Interpersonal alienation, IA (EDI-3)	44	8.5	5.67	0–20 (20)
Interoceptive deficits, ID (EDI-3)	44	11.05	9.62	0–36 (36)
Emotional dysregulation, ED (EDI-3)	44	5.84	5.46	0–23 (23)
Perfectionism, P (EDI-3)	44	7.91	4.96	0–21 (21)
Asceticism, AS (EDI-3)	44	4.89	5.43	0–24 (24)
Maturity fears, MF (EDI-3)	44	15.77	5.93	7–31 (24)
Ineffectiveness Composite, IC (EDI-3)	44	16.59	11.6	0–39 (39)
Interpersonal Problems Composite, IPC (EDI-3)	44	19.25	10.38	1–39 (38)
Affective Problems Composite, APC (EDI-3)	44	16.89	13.7	0–48 (48)
Overcontrol Composite, OC (EDI-3)	44	12.8	9.22	1–38 (37)
General Psychological Maladjustment Composite, GPMC (EDI-3)	44	81.3	43.22	19–195 (176)

**Table 2:** Body image disturbance by body parts.

Global	cm, mean (SD)	+11.44 (8.78)
	cm, range	−14.5 to +33.17
	%, mean (SD)	+45.24 (33.38)
	%, range	−33.03 to +129.69
Shoulders	cm, mean (SD)	+7.02 (10.42)
	cm, range	−31 to +30
	%, mean (SD)	+20.17 (26.53)
	%, range	−44 to +94
Chest	cm, mean (SD)	+13.25 (10.37)
	cm, range	−18 to +40
	%, mean (SD)	+53.52 (41.46)
	%, range	−41 to +182
Waist	cm, mean (SD)	+13.55 (9.68)
	cm, range	−13 to +38
	%, mean (SD)	+61.17 (43.71)
	%, range	−41 to +170
Hips	cm, mean (SD)	+11.3 (9.5)
	cm, range	−9 to +33
	%, mean (SD)	+38.54 (33.64)
	%, range	−24 to +125
Thighs	cm, mean (SD)	+12.23 (9.15)
	cm, range	−9 to +37
	%, mean (SD)	+43.01 (33.02)
	%, range	−24 to 128
Calves	cm, mean (SD)	+11.32 (9.2)
	cm, range	−7 to +35
	%, mean (SD)	+55.02 (45.97)
	%, range	−25 to +171

**Table 3:** Body image disturbance by sample groups.

		N = 53 (100%)	Relative BID (Group Mean %, SD)
Global	Underestimators (n, %)	2 (3.8)	−17.14 (22.47)
	Slight overestimators (n, %)	17 (32.1)	+19.71 (8.46)
	Great overestimators (n, %)	34 (64.2)	+61.67 (29.26)
Shoulders	Underestimators (n, %)	12 (22.6)	−13.73 (15.71)
	Slight overestimators (n, %)	25 (47.2)	+17.24 (7.15)
	Great overestimators (n, %)	16 (30.2)	+50.16 (16.88)
Chest	Underestimators (n, %)	4 (7.5)	−18.81 (16.13)
	Slight overestimators (n, %)	11 (20.8)	+21.72 (4.96)
	Great overestimators (n, %)	38 (71.7)	+61.17 (43.72)
Waist	Underestimators (n, %)	3 (5.7)	−17.89 (20.74)
	Slight overestimators (n, %)	5 (9.4)	+22.43 (5.56)
	Great overestimators (n, %)	38 (72.7)	+70.75 (39.56)
Hips	Underestimators (n, %)	4 (7.5)	−11.57 (13.36)
	Slight overestimators (n, %)	18 (34)	+15.2 (6.9)
	Great overestimators (n, %)	31 (58.5)	+58.55 (29)
Thighs	Underestimators (n, %)	3 (5.7)	−12.93 (10.23)
	Slight overestimators (n, %)	15 (28.3)	+16.88 (9.2)
	Great overestimators (n, %)	35 (66)	+59 (28.04)
Calves	Underestimators (n, %)	5 (9.4)	−12.22 (10.49)
	Slight overestimators (n, %)	9 (17)	+17.12 (9.84)
	Great overestimators (n, %)	39 (73.6)	+72.39 (40.19)

**Table 4:** Summary of results guided by hypotheses.

Main hypotheses		
(a) Relatively high prevalences of BID and/or BDS will be generally found in sisters overall	Partially accepted	Descriptive analyses of BDI values (mean, SD) support hypothesis regarding BDI: Sisters showed a mean global absolute BID of +11.44 cm (SD 8.78) and mean global relative BID of +45.24% (SD 33.38). They estimated their body size to be 11.44 cm (45.24%) larger than it actually was Descriptive analyses of BDS values (mean, SD) do not support hypothesis regarding BDS: Sisters obtained a mean BDS score of 12.11 (SD 8.88) measured by the EDI-3, which falls within the typical range for non-clinical females
(b) BDS will be positively related to BID and, since it is supposed to reflect the affective component of body image, BDS might mediate BID and affective phenomena (trait anxiety, depressive symptoms)	Partially accepted	Pearson Correlation Coefficient between BDS and: (a) BID expressed in absolute terms (r = 0.164, *p* = 0.287) and (b) BID expressed in relative terms (r = 0.157, *p* = 0.308) do not support hypothesis. Pearson Correlation Coefficient between BDS and BDI/CDI score (r = 0.604, *p* < 0.001) supports hypothesis for the case of depressive symptoms, without a mediating effect. Same testing for STAI/STAIC score (*p* > 0.05) does not support hypothesis for the case of trait anxiety
Exploratory hypotheses		
(c) A subgroup of sisters might show an atypical BID pattern potentially protective from eating and body image disorders, e.g., lower levels of BID (accurate estimation) or even inverse pattern BID (underestimation)	Accepted	Descriptive analyses of BDI values (mean, SD) support hypothesis: 17 sisters (32.1%) showed lower levels of BID close to accurate estimation; nonetheless, estimating their mean body shape to be +19.71 cm (8.46%) larger than it actually was A small minority of 2 sisters (3.8%) were global underestimators, estimating their mean body shape to be −17.14 cm (22.47%) smaller than it actually was
(d) Certain sociodemographic (age), anthropometric (BMI), lifestyle (physical activity) and/or psychological (perfectionism, trait anxiety, depressive symptoms) variables will be associated with BID and/or BDS in this population	Partially accepted	Pearson Correlation Coefficient between BID and age, BID and BMI, BDI and STAI/STAIC score. BDS and age, BDS and BMI, and BDS and STAI/STAIC score do not support hypothesis for the case of sociodemographic (age), anthropometric (BMI) and psychological variable trait anxiety (STAI/STAIC score) (*p* > 0.05) Pearson Correlation Coefficient between BID and total PAQ score (r = 0.425, *p* = 0.004) supports hypothesis for the case of lifestyle variable physical activity Pearson Correlation Coefficient between BDS and BDI/CDI score (r = 0.604, *p* < 0.001) supports hypothesis for the case of psychological variable depressive symptoms Pearson Correlation Coefficient between BDS and CAPS self-oriented perfectionism score (r = 0.427, *p* = 0.004) supports hypothesis for the case of psychological variable perfectionism Mean comparison (T-test) between BID expressed as an ordinal variable and ED, EDI-3 score (F = 3.412, *p* = 0.043, η^2^ size effect, point estimate = 0.143, 95% CI [<0.001, 0.315]) supports hypothesis for the case of psychological variable emotional dysregulation

## Data Availability

The data presented in this study are available on request from the corresponding author if intended to be used for justified academic and scientific purposes.

## Abbreviations

The following abbreviations are used in this manuscript:

BID	Body Image Disturbance
BDS	Body Dissatisfaction
BMI	Body Mass Index
AN/BN	Anorexia Nervosa/Bulimia Nervosa
m	Mean
SD	Standard Deviation
95% CI	Confidence Interval (95%)

## References

[1] Maon I., Horesh D., Gvion Y. (2020). Siblings of Individuals with Eating Disorders: A Review of the Literature. Front. Psychiatry.

[2] Heneghan A., Manitsa I., Livanou M., Treasure J. (2024). The Experiences of Having a Sibling with an Eating Disorder: A Systematic Review of the Literature. Eur. Eat. Disord. Rev..

[3] Tasa-Vinyals E., Plana M.T., Martínez-Pinteño A., Mora-Porta M., Rodríguez-Rey A., Andrés-Perpiñá S., Moreno E., Martínez E., Castro-Fornieles J., Flamarique I. (2026). Psychopathology and Other Mental Health Challenges in Siblings of Patients with Child- or Adolescent-Onset Anorexia Nervosa: A Systematic Review with a Sex/Gender Perspective. J. Clin. Med..

[4] Ghosh A., Choudhury S. (2025). Understanding Different Types of Review Articles: A Primer for Early Career Researchers. Indian J. Psychiatry.

[5] Grant M.J., Booth A. (2009). A Typology of Reviews: An Analysis of 14 Review Types and Associated Methodologies. Health Inf. Libr. J..

[6] Tasa-Vinyals E. (2018). El Espejo Subjetivo: ¿Qué Es La Imagen Corporal? | The Subjective Mirror: What Is Body Image?. Psicosom. Psiquiatr..

[7] Gila A., Castro J., Toro J., Salamero M. (1998). Subjective Body-Image Dimensions in Normal and Anorexic Adolescents. Br. J. Med. Psychol..

[8] Vankerckhoven L., Raemen L., Claes L., Eggermont S., Palmeroni N., Luyckx K. (2023). Identity Formation, Body Image, and Body-Related Symptoms: Developmental Trajectories and Associations Throughout Adolescence. J. Youth Adolesc..

[9] Tasa-Vinyals E. (2018). Mecanismos, Determinantes y Funciones de La Imagen Corporal y La (in)Satisfacción Corporal | Mechanisms, Determinants and Functions of Body Image and Body (Dis)Satisfaction. Psicosom. Psiquiatr..

[10] Tort-Nasarre G., Pocallet M.P., Artigues-Barberà E. (2021). The Meaning and Factors That Influence the Concept of Body Image: Systematic Review and Meta-Ethnography from the Perspectives of Adolescents. Int. J. Environ. Res. Public Health.

[11] Hamamoto Y., Suzuki S., Sugiura M. (2022). Two Components of Body-Image Disturbance Are Differentially Associated with Distinct Eating Disorder Characteristics in Healthy Young Women. PLoS ONE.

[12] Deek M.R., Kemps E., Prichard I. (2025). The Role of Female Family Members in Relation to Body Image and Eating Behaviour: A Cross-National Comparison between Western and Middle-Eastern Cultures. Body Image.

[13] Yager Z., Diedrichs P.C., Drummond M. (2013). Understanding the Role of Gender in Body Image Research Settings: Participant Gender Preferences for Researchers and Co-Participants in Interviews, Focus Groups and Interventions. Body Image.

[14] Merino M., Tornero-Aguilera J.F., Rubio-Zarapuz A., Villanueva-Tobaldo C.V., Martín-Rodríguez A., Clemente-Suárez V.J. (2024). Body Perceptions and Psychological Well-Being: A Review of the Impact of Social Media and Physical Measurements on Self-Esteem and Mental Health with a Focus on Body Image Satisfaction and Its Relationship with Cultural and Gender Factors. Healthcare.

[15] Deek M.R., Kemps E., Prichard I. (2024). My Mother, Sisters, and I: Investigating the Role of Female Family Members in Body Dissatisfaction and Disordered Eating Behaviours among Young Middle-Eastern Women. Body Image.

[16] Deek M.R., Prichard I., Kemps E. (2023). The Mother-Daughter-Sister Triad: The Role of Female Family Members in Predicting Body Image and Eating Behaviour in Young Women. Body Image.

[17] Chae H. (2022). Factors Associated with Body Image Perception of Adolescents. Acta Psychol..

[18] Jung E.-H., Jun M.-K. (2022). Factors Affecting Body Image Distortion in Adolescents. Children.

[19] Fabian L.J., Thompson J.K. (1989). Body Image and Eating Disturbance in Young Females. Int. J. Eat. Disord..

[20] Aleksandrova R.V., Meshkova T.A. (2025). Secular Trends in the Problems of Eating Behavior in Adolescent Girls of the Non-Clinical Population: Comparison of the Results of the Survey 2009–2011 and 2021/2023 | Дoлгoсрoчные Тенденции в Прoблемах Пищевoгo Пoведения у Девoчек-Пoдрoсткoв Неклиническoй пoпуляции: сравнение результатoв анкетирoвания 2009–2011 и 2021/2023 гoдoв. Clin. Psychol. Spec. Educ..

[21] Mora M., Penelo E., Gutiérrez T., Espinoza P., González M.L., Raich R.M. (2015). Assessment of Two School-Based Programs to Prevent Universal Eating Disorders: Media Literacy and Theatre-Based Methodology in Spanish Adolescent Boys and Girls. Sci. World J..

[22] Wilksch S.M., Wade T.D. (2009). Reduction of Shape and Weight Concern in Young Adolescents: A 30-Month Controlled Evaluation of a Media Literacy Program. J. Am. Acad. Child Adolesc. Psychiatry.

[23] Paraskeva N., Haywood S., Anquandah J., White P., Budhraja M., Diedrichs P.C., Williamson H. (2025). Evaluating the Effectiveness of a Roblox Video Game (Super U Story) in Improving Body Image Among Children and Adolescents in the United States: Randomized Controlled Trial. J. Med. Internet Res..

[24] Yager Z., Diedrichs P.C., Ricciardelli L.A., Halliwell E. (2013). What Works in Secondary Schools? A Systematic Review of Classroom-Based Body Image Programs. Body Image.

[25] Stice E., Marti C.N., Shaw H., Rohde P. (2019). Meta-Analytic Review of Dissonance-Based Eating Disorder Prevention Programs: Intervention, Participant, and Facilitator Features That Predict Larger Effects. Clin. Psychol. Rev..

[26] Jungbauer J., Heibach J., Urban K. (2016). Experiences, Burdens, and Support Needs in Siblings of Girls and Women with Anorexia Nervosa: Results from a Qualitative Interview Study. Clin. Soc. Work J..

[27] Lewis-Smith H., Diedrichs P.C., Halliwell E. (2019). Cognitive-Behavioral Roots of Body Image Therapy and Prevention. Body Image.

[28] Halliwell E., Diedrichs P.C. (2014). Testing a Dissonance Body Image Intervention among Young Girls. Health Psychol..

[29] Yetsenga R., Banerjee R., Streatfeild J., McGregor K., Austin S.B., Lim B.W.X., Diedrichs P.C., Greaves K., Mattei J., Puhl R.M. (2024). The Economic and Social Costs of Body Dissatisfaction and Appearance-Based Discrimination in the United States. Eat. Disord..

[30] Raich R.M., Sánchez-Carracedo D., López-Guimerà G., Portell M., Moncada A., Fauquet J. (2008). A Controlled Assessment of School-Based Preventive Programs for Reducing Eating Disorder Risk Factors in Adolescent Spanish Girls. Eat. Disord..

[31] Golan M., Hagay N., Tamir S. (2013). The Effect of “in Favor of Myself”: Preventive Program to Enhance Positive Self and Body Image among Adolescents. PLoS ONE.

[32] Plana M.T., Flamarique I., Julià L., Tasa-Vinyals E., Citoler B., Díaz C., Moreno E., Andrés-Perpiñá S., Martínez E., Lázaro L. (2024). Accuracy of Estimating Self and Other Body Size among Adolescent Girls with Anorexia Nervosa. Eat. Disord..

[33] Rodríguez-Rey A., Piazza-Suprani F., Tasa-Vinyals E., Plana M.T., Flamarique I., Primé-Tous M., Moreno E., Hilker I., Pujal E., Martínez E. (2025). Traumatic Events and Post-Traumatic Stress Disorder in Adolescents with Severe Eating Disorder Admitted to a Day Care Hospital. Nutrients.

[34] Tatangelo G., McCabe M., Mellor D., Mealey A. (2016). A Systematic Review of Body Dissatisfaction and Sociocultural Messages Related to the Body among Preschool Children. Body Image.

[35] Ricciardelli L.A., McCabe M.P. (2001). Children’s Body Image Concerns and Eating Disturbance: A Review of the Literature. Clin. Psychol. Rev..

[36] Johnson E.L., Salafia E.H.B. (2022). Mediating Effects of Intimacy Between Body Talk and Girls’ Body Dissatisfaction: The Forgotten Sibling Relationship. J. Youth Adolesc..

[37] Bonfanti R.C., Melchiori F., Teti A., Albano G., Raffard S., Rodgers R., Lo Coco G. (2025). The Association between Social Comparison in Social Media, Body Image Concerns and Eating Disorder Symptoms: A Systematic Review and Meta-Analysis. Body Image.

[38] Franko D.L., Fuller-Tyszkiewicz M., Rodgers R.F., Holmqvist Gattario K., Frisén A., Diedrichs P.C., Ricciardelli L.A., Yager Z., Smolak L., Thompson-Brenner H. (2015). Internalization as a Mediator of the Relationship between Conformity to Masculine Norms and Body Image Attitudes and Behaviors among Young Men in Sweden, US, UK, and Australia. Body Image.

[39] De Jesus A.Y., Ricciardelli L.A., Frisén A., Smolak L., Yager Z., Fuller-Tyszkiewicz M., Diedrichs P.C., Franko D., Gattario K.H. (2015). Media Internalization and Conformity to Traditional Masculine Norms in Relation to Body Image Concerns among Men. Eat. Behav..

[40] Gila A., Castro J., Cesena J., Toro J. (2005). Anorexia Nervosa in Male Adolescents: Body Image, Eating Attitudes and Psychological Traits. J. Adolesc. Health.

[41] Toro J., Gila A., Castro J., Pombo C., Guete O. (2005). Body Image, Risk Factors for Eating Disorders and Sociocultural Influences in Spanish Adolescents. Eat. Weight Disord.-Stud. Anorex. Bulim. Obes..

[42] Gualdi-Russo E., Rinaldo N., Zaccagni L. (2022). Physical Activity and Body Image Perception in Adolescents: A Systematic Review. Int. J. Environ. Res. Public Health.

[43] Carballo Afonso R., Diz Gómez J.C., Redondo Gutiérrez L., Ayán Pérez C. (2023). Influence of Exercise in the Body Image of Preadolescents and Adolescents: Importance of the Index of Corporal Mass as a Factor of Confusion. Nutr. Hosp..

[44] Li Q., Li H., Zhang G., Cao Y., Li Y. (2024). Athlete Body Image and Eating Disorders: A Systematic Review of Their Association and Influencing Factors. Nutrients.

[45] Fatt S.J., George E., Hay P., Jeacocke N., Gotkiewicz E., Mitchison D. (2024). An Umbrella Review of Body Image Concerns, Disordered Eating, and Eating Disorders in Elite Athletes. J. Clin. Med..

[46] American Psychiatric Association (2022). Diagnostic and Statistical Manual of Mental Disorders.

[47] Van Eeden A.E., Van Hoeken D., Hoek H.W. (2021). Incidence, Prevalence and Mortality of Anorexia Nervosa and Bulimia Nervosa. Curr. Opin. Psychiatry.

[48] Hebebrand J., Bulik C.M. (2011). Critical Appraisal of the Provisional DSM-5 Criteria for Anorexia Nervosa and an Alternative Proposal. Int. J. Eat. Disord..

[49] American Psychiatric Association (2000). Diagnostic and Statistical Manual of Mental Disorders.

[50] Karakuş Aydos Y., Dövencioğlu D., Karlı Oğuz K., Özdemir P., Pehlivantürk Kızılkan M., Kanbur N., Ünal D., Nalbant K., Çetin Çuhadaroğlu F., Akdemir D. (2024). Neural Correlates of Distorted Body Images in Adolescent Girls with Anorexia Nervosa: How Is It Different from Major Depressive Disorder?. J. Neuropsychol..

[51] Esposito R., Cieri F., di Giannantonio M., Tartaro A. (2018). The Role of Body Image and Self-Perception in Anorexia Nervosa: The Neuroimaging Perspective. J. Neuropsychol..

[52] Himmerich H., Bentley J., Kan C., Treasure J. (2019). Genetic Risk Factors for Eating Disorders: An Update and Insights into Pathophysiology. Ther. Adv. Psychopharmacol..

[53] Bulik C.M., Sullivan P.F., Tozzi F., Furberg H., Lichtenstein P., Pedersen N.L. (2006). Prevalence, Heritability, and Prospective Risk Factors for Anorexia Nervosa. Arch. Gen. Psychiatry.

[54] Holland A.J., Hall A., Murray R., Russell G.F., Crisp A.H. (1984). Anorexia Nervosa: A Study of 34 Twin Pairs and One Set of Triplets. Br. J. Psychiatry.

[55] Benninghoven D., Tetsch N., Jantschek G. (2008). Patients with Eating Disorders and Their Siblings: An Investigation of Body Image Perceptions. Eur. Child Adolesc. Psychiatry.

[56] Amianto F., Abbate-Daga G., Morando S., Sobrero C., Fassino S. (2011). Personality Development Characteristics of Women with Anorexia Nervosa, Their Healthy Siblings and Healthy Controls: What Prevents and What Relates to Psychopathology?. Psychiatry Res..

[57] Steinhausen H.C., Jakobsen H., Helenius D., Munk-Jørgensen P., Strober M. (2015). A Nation-Wide Study of the Family Aggregation and Risk Factors in Anorexia Nervosa over Three Generations. Int. J. Eat. Disord..

[58] Maloney M.J., Phyllis S.-S.W. (1983). Eating Attitudes and Behaviors of Anorexia Nervosa Patients and Their Sisters. Gen. Hosp. Psychiatry.

[59] Phillipou A., Rossell S.L., Castle D.J., Gurvich C. (2022). Interoceptive Awareness in Anorexia Nervosa. J. Psychiatr. Res..

[60] Degortes D., Zanetti T., Tenconi E., Santonastaso P., Favaro A. (2014). Childhood Obsessive-Compulsive Traits in Anorexia Nervosa Patients, Their Unaffected Sisters and Healthy Controls: A Retrospective Study. Eur. Eat. Disord. Rev..

[61] Castro-Fornieles J., Gual P., Lahortiga F., Gila A., Casulà V., Fuhrmann C., Imirizaldu M., Saura B., Martínez E., Toro J. (2007). Self-Oriented Perfectionism in Eating Disorders. Int. J. Eat. Disord..

[62] Papero D.V. (1990). Bowen Family Systems Theory.

[63] Harris P.A., Taylor R., Minor B.L., Elliott V., Fernandez M., O’Neal L., McLeod L., Delacqua G., Delacqua F., Kirby J. (2019). The REDCap Consortium: Building an International Community of Software Platform Partners. J. Biomed. Inform..

[64] Hollingshead A. (1975). Four-Factor Index of Social Status.

[65] Kling J., Kwakkenbos L., Diedrichs P.C., Rumsey N., Frisén A., Brandão M.P., Silva A.G., Dooley B., Rodgers R.F., Fitzgerald A. (2019). Systematic Review of Body Image Measures. Body Image.

[66] Gila A., Castro J., Toro J., Salamero M. (2004). Subjective Body Image Dimensions in Normal Female Population: Evolution Through Adolescence and Early Adulthood.

[67] Garner D.M. (2004). The Eating Disorder Inventory-3 Professional Manual.

[68] Clausen L., Rosenvinge J.H., Friborg O., Rokkedal K. (2010). Validating the Eating Disorder Inventory-3 (EDI-3): A Comparison Between 561 Female Eating Disorders Patients and 878 Females from the General Population. J. Psychopathol. Behav. Assess..

[69] Garner D.M., Elosua P., López-Jáuregui A., Sánchez-Sánchez F. (2010). EDI 3. Inventario de Trastornos de La Conducta Alimentaria-3. Manual.

[70] Garner D.M. (1983). Development and Validation of a Multidimensional Eating Disorder Inventory for Anorexia Nervosa and Bulimia. Int. J. Eat. Disord..

[71] Janz K.F., Lutuchy E.M., Wenthe P., Levy S.M. (2008). Measuring Activity in Children and Adolescents Using Self-Report: PAQ-C and PAQ-A. Med. Sci. Sports Exerc..

[72] Martínez-Gómez D., Martínez-De-Haro V., Pozo T., Welk G.J., Villagra A., Calle M.E., Marcos A., Veiga O.L. (2009). Reliability and Validity of the PAQ-A Questionnaire to Assess Physical Activity in Spanish Adolescents. Rev. Esp. Salud Publica.

[73] Martínez-Lemos R., Pérez C.A., Lastra A.S., Carral J.M.C., Sánchez R.V. (2016). Physical Activity Questionnaires for Spanish Children and Adolescents: A Systematic Review. An. Sist. Sanit. Navar..

[74] Marasso D., Lupo C., Collura S., Rainoldi A., Brustio P.R. (2021). Subjective versus Objective Measure of Physical Activity: A Systematic Review and Meta-Analysis of the Convergent Validity of the Physical Activity Questionnaire for Children (PAQ-C). Int. J. Environ. Res. Public Health.

[75] Rostami Haji Abadi M., Kwaghbo N., Onyeso O.K., Sadia F., Benavides Castro J.K., Silva D.A.S., Yan Y., Shen Y., Hamrik Z., Sarmiento O.L. (2025). Psychometric Properties of Physical Activity Questionnaires for Children and Adolescents: An Updated Systematic Review. Sports Med..

[76] Benítez-Porres J., Alvero-Cruz J.R., Sardinha L.B., López-Fernández I., Carnero E.A. (2016). Cut-off Values for Classifying Active Children and Adolescents Using the Physical Activity Questionnaire: PAQ-C and PAQ-A. Nutr. Hosp..

[77] Flett G.L., Hewitt P.L., Besser A., Su C., Vaillancourt T., Boucher D., Munro Y., Davidson L.A., Gale O. (2016). The Child–Adolescent Perfectionism Scale. J. Psychoeduc. Assess..

[78] Vicent M., Inglés C.J., Sanmartín R., Gonzálvez C., Delgado B., García-Fernández J.M. (2019). Spanish Validation of the Child and Adolescent Perfectionism Scale: Factorial Invariance and Latent Means Differences across Sex and Age. Brain Sci..

[79] Castro J., Gila A., Gual P., Lahortiga F., Saura B., Toro J. (2004). Perfectionism Dimensions in Children and Adolescents with Anorexia Nervosa. J. Adolesc. Health.

[80] Spielberger C.D., Edwards C.D., Montouri J., Lushene R. (1973). State-Trait Anxiety Inventory for Children.

[81] Spielberger C.D. (2012). State-Trait Anxiety Inventory for Adults.

[82] Bieling P.J., Antony M.M., Swinson R.P. (1998). The State–Trait Anxiety Inventory, Trait Version: Structure and Content Re-Examined. Behav. Res. Ther..

[83] Beckler K. (2010). State-Trait Anxiety Inventory for Adults Sampler Set Manual, Instrument and Scoring Guide. 1983 Consulting.

[84] Buela-Casal G., Guillén-Riquelme A., Seisdedos Cubero N. (1986). Inventario de Ansiedad Estado-Rasgo.

[85] Spielberger C., Gonzalez-Reigosa F., Martinez-Urrutia A., Natalicio L., Natalicio D.S. (1971). Development of the Spanish Edition of the State-Trait Anxiety Inventory. Interam. J. Psychol..

[86] Riquelme A.G. (2014). Validación de La Adaptación Española Del State-Trait Anxiety Inventory En Diferentes Muestras Españolas. Doctoral Dissertation.

[87] Beck A.T., Ward C.H., Mendelson M., Mock J., Erbaugh J. (1961). An Inventory for Measuring Depression. Arch. Gen. Psychiatry.

[88] Kovacs M. (1985). The Children’s Depression, Inventory (CDI). Psychopharmacol. Bull..

[89] Sanz J., Luis A., Carmelo P., Resumen V. (2003). Adaptación Española Del Inventario Para La Depresión de Beck-II (BDI-II): 2. Propiedades Psicométricas En Población General. Clin. Salud.

[90] Masip A.F., Amador-Campos J.A., Gómez-Benito J., Gándara V.D.B. (2010). Psychometric Properties of the Children’s Depression Inventory in Community and Clinical Sample. Span. J. Psychol..

[91] Davanzo P., Kerwin L., Nikore V., Esparza C., Forness S., Murrelle L. (2004). Spanish Translation and Reliability Testing of the Child Depression Inventory. Child Psychiatry Hum. Dev..

[92] Tovée M.J., Benson P.J., Emery J.L., Mason S.M., Cohen-Tovée E.M. (2003). Measurement of Body Size and Shape Perception in Eating-Disordered and Control Observers Using Body-Shape Software. Br. J. Psychol..

[93] Costantini I., Eley T.C., Pingault J.-B., Davies N.M., Bould H., Bulik C.M., Krebs G., Lewis G., Lewis G., Llewellyn C. (2026). Longitudinal Associations between Adolescent Body Dissatisfaction, Eating Disorder and Depressive Symptoms, and BMI: A UK Twin Cohort Study. Lancet Psychiatry.

[94] Penelo E., Espinoza P., Portell M., Raich R.M. (2012). Assessment of Body Image: Psychometric Properties of the Body Image Questionnaire. J. Health Psychol..

[95] Stern J.M. (2018). Transcultural Aspects of Eating Disorders and Body Image Disturbance‡. Nord. J. Psychiatry.

[96] Castro J., Toro J., Salamero M., Guimerá E. (1991). The Eating Attitudes Test: Validation of the Spanish Version. Eval. Psicol..

[97] Francisco R., Espinoza P., González M.L., Penelo E., Mora M., Rosés R., Raich R.M. (2015). Body Dissatisfaction and Disordered Eating among Portuguese and Spanish Adolescents: The Role of Individual Characteristics and Internalisation of Sociocultural Ideals. J. Adolesc..

[98] Areemit R.S., Katzman D.K., Pinhas L., Kaufman M.E. (2010). The Experience of Siblings of Adolescents With Eating Disorders. J. Adolesc. Health.

[99] Haraway D.J. (1991). Simians, Cyborgs and Women: The Reinvention of Nature.

[100] Lykke N. (2010). Feminist Studies: A Guide to Intersectional Theory, Methodology and Writing.

[101] Golden N.H., Walsh B.T. (2024). Time to Revisit the Definition of Atypical Anorexia Nervosa. Int. J. Eat. Disord..

[102] Golden N.H., Mehler P.S. (2020). Atypical Anorexia Nervosa Can Be Just as Bad. Clevel. Clin. J. Med..

[103] Walsh B.T., Hagan K.E., Lockwood C. (2023). A Systematic Review Comparing Atypical Anorexia Nervosa and Anorexia Nervosa. Int. J. Eat. Disord..

[104] Sawyer S.M., Whitelaw M., Le Grange D., Yeo M., Hughes E.K. (2016). Physical and Psychological Morbidity in Adolescents with Atypical Anorexia Nervosa. Pediatrics.

